# Comorbidity network of post-traumatic stress and depressive symptoms during the COVID-19 pandemic in Korea

**DOI:** 10.4178/epih.e2026006

**Published:** 2026-01-23

**Authors:** Yujin Lee, Ji Su Yang, Alexander C. Tsai, Jee In Kang, Hearan Koo, Hyeon Woo Yim, Hyeon Chang Kim, Sun Jae Jung

**Affiliations:** 1 Department of Preventive Medicine, Yonsei University College of Medicine, Seoul, Korea; 2 Center for Global Health, Massachusetts General Hospital, Boston, MA, USA; 3 Harvard Medical School, Boston, MA, USA; 4 Department of Epidemiology, Harvard T.H. Chan School of Public Health, Boston, MA, USA; 5 Department of Psychiatry, Yonsei University College of Medicine, Seoul, Korea; 6 Institute of Behavioral Science in Medicine, Yonsei University College of Medicine, Seoul, Korea; 7 The Korea Social Science Data Archive, Seoul National University, Seoul, Korea; 8 Department of Preventive Medicine, College of Medicine, The Catholic University, Seoul, Korea; 9 Cardiovascular and Metabolic Diseases Etiology Research Center, Yonsei University College of Medicine, Seoul, Korea; 10 Institute for Innovation in Digital Healthcare, Yonsei University, Seoul, Korea

**Keywords:** Social network analysis, Stress disorders, post-traumatic, Depression, Sleep wake disorders, COVID-19

## Abstract

**OBJECTIVES:**

The coronavirus disease 2019 (COVID-19) pandemic had direct effects on population health through infection and morbidity, as well as indirect effects on population mental health. We estimated the network structure of post-traumatic stress symptoms (PTSS) and depressive symptoms throughout the pandemic in Korea and aimed to identify the most central and bridging symptoms.

**METHODS:**

Participants aged 30–64 years completed mental health surveys across 3 phases of the COVID-19 pandemic: March 2020 (n=1,925), February–March 2021 (n=1,754), and December 2021–January 2022 (n=1,595). Using PTSS and depressive symptom data, we conducted network analyses, and the primary measures of symptom importance (centrality) were expected influence and bridge expected influence.

**RESULTS:**

In the comorbidity network, although the most central symptoms fluctuated over the course of the pandemic, sleep problems were consistently identified as the most influential bridge symptoms throughout. The symptom network structure differed between the subacute and chronic phases of the pandemic.

**CONCLUSIONS:**

We found evidence of changes in the network structure of PTSS and depressive symptoms, even as sleep problems retained a consistent role as a bridging symptom. Although overall network structures varied across phases of the pandemic, the bridging role of sleep-related symptoms remained consistently strong, suggesting that sleep problems may represent a general and enduring mechanism underlying PTSS–depression comorbidity. During future pandemics, prompt screening for sleep problems may help prevent the development of comorbidity between PTSS and depressive symptoms.

## GRAPHICAL ABSTRACT

[Fig f5-epih-48-e2026006]

## Key Message

• The comorbidity network between PTSS and depressive symptoms shifted from the subacute to the chronic phase of the COVID-19 pandemic, with changes in central symptoms.

• “Flashbacks” and “depressed mood” were central in the subacute phase, whereas “depressed mood” and “reckless behavior” were central in the chronic phase.

• “Sleep problems” consistently acted as a key bridge symptom, suggesting its potential as an intervention target.

## INTRODUCTION

The coronavirus disease 2019 (COVID-19) pandemic was associated with post-traumatic stress symptoms (PTSS) resulting from fear, uncertainty, and bereavement caused by rapidly increasing infections, hospitalizations, and deaths, as well as from government-imposed restrictions (“lockdowns”) and self-imposed social distancing [1]. Consistent with these observations, the COVID-19 pandemic has been conceptualized as a mass or collective trauma that triggered a wide range of psychological distress symptoms, including anxiety, depression, and PTSS [2-5]. These symptoms of psychological distress were more broadly disseminated across the population than COVID-19 infection itself [6]. Several meta-analyses of general population data collected during the early stages of the COVID-19 pandemic have reported prevalence estimates of clinically significant psychiatric symptoms ranging from 15% to 20% [7]. Among individuals with PTSS, studies have estimated rates of comorbidity with depressive symptoms ranging from 21% to 94% [8-11].

Longitudinal studies have found that PTSS and depressive symptoms tend to co-vary [12]. As the pandemic has continued, many individuals have adapted to its prolonged societal impacts, which may reduce awareness of pandemic-related trauma. However, the psychological effects of COVID-19 at the population level may persist and continue to manifest in different patterns over time. For example, a longitudinal study of a general population sample in China reported persistently high PTSS despite a significant reduction in overall psychological impact [13]. Additionally, a meta-analysis of longitudinal studies found that psychological symptoms increased slightly during the early phases of COVID-19, whereas symptom severity declined significantly during subsequent phases [14]. Nevertheless, relatively little research has focused on changes in the network structure of PTSS and depressive symptoms across different stages of the pandemic.

Notably, large-scale symptom network studies examining comorbid PTSS and depressive symptoms in Korean populations remain scarce. The present study addresses this gap by examining symptom-level dynamics across multiple phases of the COVID-19 pandemic. In particular, this study is distinguished from prior work by employing a repeated cross-sectional design to compare symptom network structures across different pandemic phases and by drawing on a large, community-based Korean sample. Symptom network analysis conceptualizes mental disorders as networks of interacting psychological symptoms rather than as latent constructs that give rise to symptoms [15,16]. From a clinical perspective, this approach may help identify central symptoms for targeted intervention and bridge symptoms that contribute to the propagation of comorbidity [17]. Although prior studies have examined comorbidity between PTSS and depressive symptoms, important gaps remain in understanding symptom-level interactions throughout the COVID-19 pandemic. Therefore, we aimed to assess interactions between PTSS and depressive symptoms across pandemic phases and to identify central and bridge symptoms using network analysis in the general population. Specifically, we first aimed to identify the most influential symptoms in the comorbidity network across the pandemic. Second, we sought to determine which symptoms accounted for observed associations within the comorbid structure during each pandemic phase. Finally, we statistically compared results across phases.

## MATERIALS AND METHODS

### Data collection and participants

**Figure 1.**
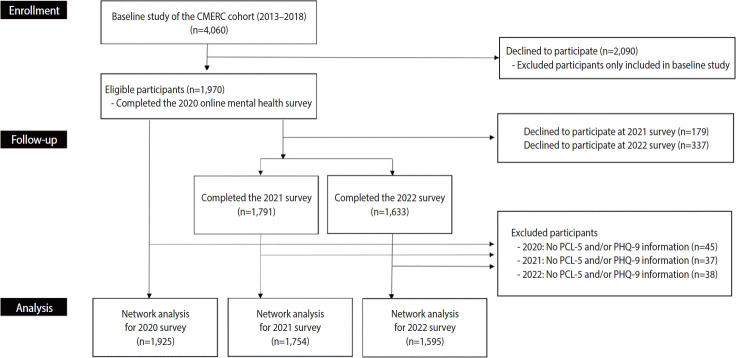
Flow diagram of study participants for network analysis. CMERC, Cardiovascular and Metabolic Etiology Research Center; PCL-5, Post-traumatic Stress Disorder Checklist for the Diagnostic and Statistical Manual of Mental Disorders, 5th edition; PHQ-9, Patient Health Questionnaire-9.

This study utilized the Cardiovascular and Metabolic Etiology Research Center (CMERC) COVID-19 online Mental Health Survey (CC-MHS), which was conducted across multiple phases of the COVID-19 pandemic. The CMERC study was a community-based prospective cohort study conducted between 2013 and 2018 and enrolled 4,060 participants aged 30–64 years at baseline [18]. The CC-MHS was administered to CMERC cohort participants from shortly after the first COVID-19 outbreak through 2023 (Supplementary Material 1). In the present analysis, data from 3 survey waves were utilized to ensure approximately 1-year intervals between assessments. For each survey wave, we contacted 3,940 of the 4,060 baseline participants to request participation, excluding 59 individuals who had withdrawn consent or died and 61 individuals who could not be reached via mobile phone. Among respondents, individuals who did not provide information on the Post-traumatic Stress Disorder Checklist for the Diagnostic and Statistical Manual of Mental Disorders, 5th edition (DSM-5) (PCL-5) and the Patient Health Questionnaire-9 (PHQ-9) were excluded [19,20]. The number of respondents for each survey wave was as follows: (1) the 2020 survey, in which 1,925 of 1,970 respondents were included; (2) the 2021 survey, in which 1,754 of 1,791 respondents were included; and (3) the 2022 survey, in which 1,595 of 1,633 respondents completed the survey (Figure 1). Specifically, the 2020 survey was conducted within 1 month to 3 months after the COVID-19 outbreak and was defined as the “subacute phase,” whereas the 2021 and 2022 surveys were conducted more than 3 months after the outbreak and were defined as the “chronic phase.”

### Measures

#### Assessing depressive symptoms

Depressive symptoms were assessed using the self-reported PHQ-9, which contains 9 items measuring the frequency of depressive symptoms over the preceding 2 weeks [20]. Each item is rated in frequency on a 4-point scale as follows: 0=not at all, 1=several days, 2=more than half of the days, and 3=nearly every day. Scores on the PHQ-9 range from 0–27. This questionnaire has been validated for use in the Korean population [21].

#### Assessing PTSS

PTSS were assessed using the PCL-5, a 20-item questionnaire that measures PTSS severity over the past month [19]. Each item is rated on a 5-point scale ranging from 0 (not at all) to 4 (extremely), resulting in total scores ranging from 0 to 80. The PCL-5 assesses 4 symptom clusters of PTSS: intrusion, avoidance, negative alterations in cognition and mood, and hyperarousal. The PCL-5 has also been validated for use in the Korean population [22].

### Statistical analysis

We conducted the analyses in 4 steps: (1) network estimation, (2) network inference, (3) network robustness assessment, and (4) network comparison. All analyses were performed using R version 4.0.4 (R Foundation for Statistical Computing, Vienna, Austria) within the RStudio development environment (version 1.4.1106).

Symptom networks were estimated using Gaussian graphical models implemented through the R package *qgraph* [23]. In the network structure, symptoms are represented as nodes, and edges represent estimated associations between nodes. Positive associations are depicted in blue, whereas negative associations are depicted in red. Edge thickness reflects the strength of the estimated association, with thicker edges indicating stronger associations. Partial correlation networks were estimated using the least absolute shrinkage and selection operator with the Extended Bayesian Information Criterion *EBICglasso* function in the *qgraph* package [24]. In addition, because several PCL-5 and PHQ-9 items assess conceptually similar symptoms, such as sleep and concentration problems, we evaluated potential item redundancy using the *goldbricker* function from the *networktools* R package [25]. We used the expected influence (EI) metric to assess the relative importance of symptoms within the network. EI accounts for both positive and negative edge weights extending from a given node and may provide a more appropriate measure of node strength in psychopathological networks with negative edges [26]. We also calculated bridge EI to identify potential bridging symptoms between communities. Bridge EI is defined as the sum of positive and negative edge weights connecting a node to nodes outside its own community [25,26]. Centrality indices were visualized using standardized Z-scores to facilitate comparison across networks.

Network robustness was evaluated using the bootnet R package [27]. We assessed the accuracy of edge weights by computing non-parametric bootstrapped 95% confidence intervals (CIs) based on 1,000 bootstrap samples. Network stability was examined using a case-dropping bootstrap procedure to assess the stability of centrality measures. A correlation stability (CS) coefficient of ≥0.5 was considered acceptable for interpreting centrality stability [27]. We further conducted bootstrapped difference tests to compare node and edge strengths, with each test performed 1,000 times for each network.

Finally, omnibus comparison tests were conducted to examine similarities and differences between networks estimated during the subacute phase (2020 survey) and the chronic phases (2021 and 2022 surveys) [28]. We performed 3 pairwise comparisons to evaluate differences in network structure across phases. These analyses yielded p-values for M, representing differences in overall network structure, and for S, representing differences in global EI. To obtain reliable comparison results, we conducted 1,000 permutations, which corresponds to the minimum recommended number [28].

### Ethics statement

The study protocol was approved by the institutional review board of the hospital at Yonsei University College of Medicine (Y-2020-0006), and written informed consent was obtained from all participants. All procedures in this work complied with the ethical standards of the relevant national and institutional committees on human experimentation and were carried out in accordance with the Ethical Principles for Medical Research from the Helsinki Declaration of 1975 revised in 2008.

## RESULTS

We estimated 3 cross-sectional symptom networks using the 2020, 2021, and 2022 surveys. In each estimated network, the PCL-5 and PHQ-9 nodes formed 2 distinct communities. Most edges were positive, although a small number of negative edges were also observed. Overall, the sample was composed primarily of current drinkers, married individuals, participants with more than 12 years of education, those with household income levels in the second quartile, individuals without a history of disease, and those not currently taking medications (Table 1). In addition, in the 2020 survey, there were no significant differences in PHQ-9 or PCL-5 scores by gender; however, women had higher PHQ-9 and PCL-5 scores than men in the 2021 and 2022 surveys (Table 1).

**Figure 2.**
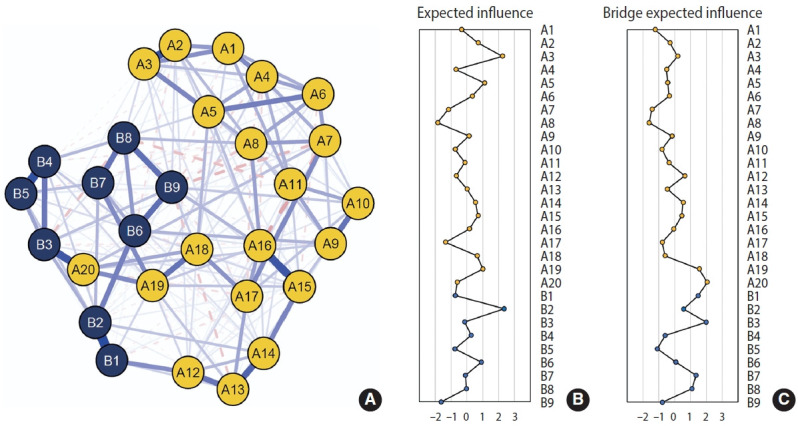
Comorbidity network structures of the 2020 survey. (A) Estimated network of PCL-5 and PHQ-9 symptoms, (B) centrality indices for the estimated comorbidity network, (C) bridge centrality indices for the estimated comorbidity network. A1–A20 are items from the PCL-5: A1: intrusive thoughts; A2: nightmares; A3: flashbacks; A4: emotional cue reactivity; A5: physiological cue reactivity; A6: avoidance of thoughts; A7: avoidance of reminders; A8: trauma-related amnesia; A9: negative beliefs; A10: blame of self or others; A11: negative trauma-related emotions; A12: loss of interest; A13: detachment; A14: restricted affect; A15: irritability/anger; A16: reckless/self-destructive behavior; A17: hypervigilance; A18: exaggerated startle response; A19: difficulty concentrating; A20: sleep disturbance. B1–B9 are items from the PHQ-9: B1: anhedonia; B2: depressed mood; B3; sleeping problems; B4: fatigability; B5: appetite problems; B6: negative feeling by myself; B7: concentration problems; B8: agitation/retardation; B9: suicidal ideation. PHQ-9, Patient Health Questionnaire-9; PCL-5, Post-traumatic Stress Disorder Checklist for the Diagnostic and Statistical Manual of Mental Disorders, fifth edition.

**Figure 3.**
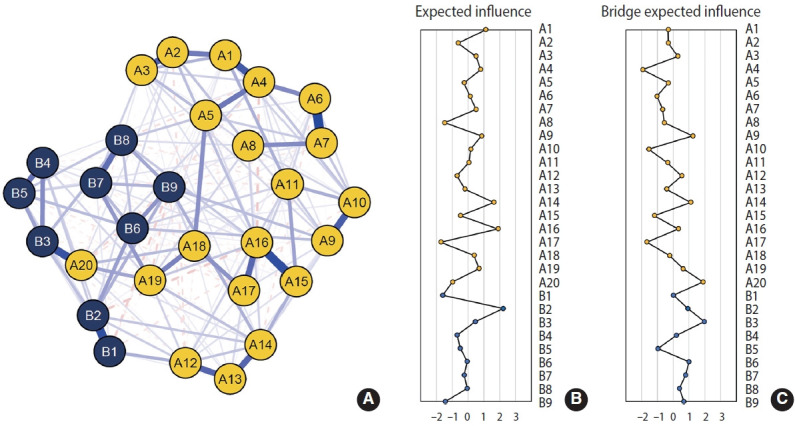
Comorbidity network structures of the 2021 survey. (A) Estimated network of PCL-5 and PHQ-9 symptoms, (B) centrality indices for the estimated comorbidity network, (C) bridge centrality indices for the estimated comorbidity network. A1-A20 are items from the PCL-5: A1: intrusive thoughts; A2: nightmares; A3: flashbacks; A4: emotional cue reactivity; A5: physiological cue reactivity; A6: avoidance of thoughts; A7: avoidance of reminders; A8: trauma-related amnesia; A9: negative beliefs; A10: blame of self or others; A11: negative trauma-related emotions; A12: loss of interest; A13: detachment; A14: restricted affect; A15: irritability/anger; A16: reckless/self-destructive behavior; A17: hypervigilance; A18: exaggerated startle response; A19: difficulty concentrating; A20: sleep disturbance. B1-B9 are items from the PHQ-9: B1: anhedonia; B2: depressed mood; B3: sleeping problems; B4: fatigability; B5: appetite problems; B6: negative feeling by myself; B7: concentration problems; B8: agitation/retardation; B9: suicidal ideation. PHQ-9, Patient Health Questionnaire-9; PCL-5, Post-traumatic Stress Disorder Checklist for the Diagnostic and Statistical Manual of Mental Disorders, fifth edition.

**Figure 4.**
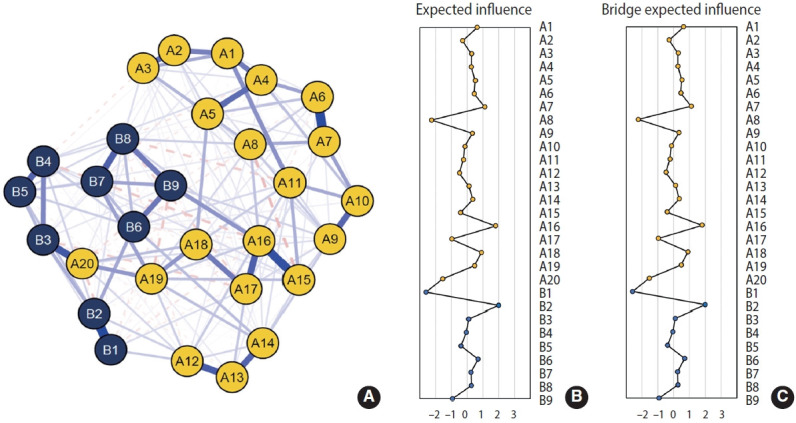
Comorbidity network structures of the 2022 survey. (A) Estimated network of PCL-5 and PHQ-9 symptoms, (B) centrality indices for the estimated comorbidity network, (C) bridge centrality indices for the estimated comorbidity network. A1-A20 are items from the PCL-5: A1: intrusive thoughts; A2: nightmares; A3: flashbacks; A4: emotional cue reactivity; A5: physiological cue reactivity; A6: avoidance of thoughts; A7: avoidance of reminders; A8: trauma-related amnesia; A9: negative beliefs; A10: blame of self or others; A11: negative trauma-related emotions; A12: loss of interest; A13: detachment; A14: restricted affect; A15: irritability/anger; A16: reckless/self-destructive behavior; A17: hypervigilance; A18: exaggerated startle response; A19: difficulty concentrating; A20: sleep disturbance. B1-B9 are items from the PHQ-9: B1: anhedonia; B2: depressed mood; B3; sleeping problems; B4: fatigability; B5: appetite problems; B6: negative feeling by myself; B7: concentration problems; B8: agitation/retardation; B9: suicidal ideation. PHQ-9, Patient Health Questionnaire-9; PCL-5, Post-traumatic Stress Disorder Checklist for the Diagnostic and Statistical Manual of Mental Disorders, fifth edition.

Figure 2A and Supplementary Material 2 present the symptom network structure estimated using the 2020 data. The edge with the largest magnitude corresponded to the association between sleep disturbance and sleeping problems (A20 and B3; edge weight=0.38). EI centrality is shown in Figure 2B. Depressed mood (B2, EI=2.35) and flashbacks (A3, EI=2.24) exhibited the highest node EI values. In contrast, trauma-related amnesia (A8, EI=−1.85) and suicidal ideation (B9, EI=−1.64) showed relatively lower node EI. Figure 2C presents bridge EI. Nodes with relatively high bridge EI values included sleep disturbance (A20, standardized bridge EI=2.04) and sleeping problems (B3, bridge EI=1.97). The symptom network structure estimated using the 2021 data is shown in Figure 3 and Supplementary Material 3, whereas the network structure estimated using the 2022 survey is shown in Figure 4 and Supplementary Material 4. Overall, the estimated network structure across all 3 years was largely similar. Detailed descriptions of the network structures for the 2020, 2021, and 2022 surveys are provided below and in Table 2.

Across all 3 annual networks (2020–2022), the edge between sleep disturbance (A20) and sleeping problems (B3) consistently represented the strongest cross-community association between PCL-5 and PHQ-9 symptoms (Table 3). Specifically, the A20–B3 edge weight was 0.38 in 2020, 0.45 in 2021, and 0.44 in 2022, ranking first among all cross-group (A–B) edges in each corresponding network. When examining connections from A20 to PHQ-9 symptoms, B3 was the most strongly associated node in all years, followed by weaker associations with B9 and B6 in 2020, B9 and B6 in 2021, and B1 and B9 in 2022. Similarly, for B3, A20 was consistently the most strongly associated PCL-5 symptom, followed by A15 and A3 in 2020, A12 and A19 in 2021, and A17 and A19 in 2022. These findings indicate that sleep-related symptoms exhibited the most robust and stable cross-community connections across all survey waves.

All 3 networks were stably estimated, with small to moderate CIs around the edge weights (Supplementary Material 5). In the stability analysis using case-dropped bootstrapping, the CS coefficients for EI were 0.75 across all networks. The CS coefficients for bridge EI were 0.67 for the 2020 survey and 0.75 for both the 2021 and 2022 surveys (Supplementary Material 6), all of which exceeded the recommended threshold.

The network comparison test indicated that the network structure estimated using the 2020 data differed from the network estimated using the 2021 data (M=0.38, p<0.01) and from the network estimated using the 2022 data (M=0.40, p<0.01). The global strength invariance tests showed no differences between the 2020 and 2021 networks (S=0.40, p=0.85) or between the 2020 and 2022 networks (S=0.10, p=0.97). When comparing the 2021 and 2022 networks, the network structure invariance test (M=0.17, p=0.37) and the global strength invariance test (S=0.30, p=0.85) indicated that the network structures did not differ.

## DISCUSSION

In this Korean general-population study with 3 serial cross-sections conducted throughout the COVID-19 pandemic, we estimated 3 symptom networks of depression and post-traumatic stress. When comparing networks across time points, the estimated network structure differed significantly between the subacute (2020 survey) and chronic (2021 and 2022 surveys) phases of the COVID-19 pandemic. In particular, as the pandemic progressed from the subacute phase to the chronic phase, the structure of the comorbidity network changed, especially with respect to the most central symptoms. Sleep problems or sleep disturbance were consistently identified as the most influential bridge symptom across comorbidity networks in all phases of the pandemic. In contrast, with regard to central symptoms, flashbacks, which were among the most central symptoms along with depressed mood in the subacute phase, were no longer among the most influential central symptoms in the chronic phase, during which depressed mood and reckless behavior emerged as the most central. From a network perspective, these findings suggest that the comorbidity network structure changed across COVID-19 phases, while the bridge symptom of sleep problems remained constant.

In our study, depressed mood was estimated to be the most central symptom. This finding is consistent with the central role of depressed mood in screening for depressive disorders based on DSM-5 criteria. After the COVID-19 outbreak, depressive symptoms associated with social distancing and shutdown measures were more prevalent than other psychological symptoms in the general population; anxiety or depressive symptoms were reported in approximately 30% to 48% of individuals, compared with PTSS, which were reported in approximately 4.6% to 26.3% of individuals [13,29-33]. Considering these findings, depressed mood may play a central role in activating and maintaining psychological distress throughout the pandemic. However, relatively little research has examined this possibility [34]. Greater understanding of depressed mood and its potential influence on overall psychological distress during subsequent chronic phases of the pandemic is therefore needed.

We observed changing patterns in PTSS symptoms across pandemic phases. Flashbacks were the most central PTSS symptom in the subacute phase of the pandemic but were no longer among the most central symptoms in the chronic phase. This finding is consistent with previous cross-sectional and longitudinal studies examining comorbid PTSS–depressive symptom networks [9,34-38]. During COVID-19 outbreaks, collective or mass trauma may generate intrusive images, including flashbacks, related to perceived infection risk; however, the intensity of such intrusive experiences may subsequently diminish. The observed changes in symptom network structure during the chronic phase may reflect increased avoidance of trauma-related triggers, as well as greater access to reliable COVID-19 information from the World Health Organization and government quarantine guidelines, and broader public adaptation to the pandemic.

With respect to comorbidity between PTSS and depressive disorders, we found that sleep problems emerged as major bridge symptoms, replicating findings from previous studies [9,34,35]. Sleep is a critical contributor to mental health, and many psychiatric diagnoses include sleep disturbance as a non-specific DSM-5 criterion [39]. In general, individuals with psychiatric disorders experience sleep difficulties at rates of approximately 50% to 80% [40], and sleep problems are closely linked to disorder onset and progression [41,42]. If sleep problems serve a key bridging function, they may facilitate cross-activation, such that poor sleep among individuals with depressive disorders increases vulnerability to post-traumatic stress disorder (PTSD), or poor sleep among individuals with PTSD increases vulnerability to depression.

Although overall network structures varied across pandemic phases, the bridging role of sleep-related symptoms remained consistently strong across all time points. This pattern suggests that the centrality of sleep problems is not merely pandemic-specific but reflects a more general and enduring mechanism underlying comorbidity between PTSS and depressive symptoms. These findings underscore the need for further consideration of sleep-focused interventions for comorbid PTSD and depression. Building on this evidence, public health–level mental health programs may benefit from actively integrating sleep hygiene education. Such sleep-focused interventions are cost-effective, scalable, and supported by robust evidence across diverse populations, suggesting that their incorporation into community-based mental health strategies could strengthen both prevention and early intervention efforts for comorbid PTSD and depression.

The strengths of this study contribute to existing knowledge in several ways. First, participants in the CC-MHS were assessed repeatedly across subacute and chronic phases of the COVID-19 pandemic, allowing for detailed evaluation of mental health patterns in the community and comparisons across pandemic stages. Second, we focused on individual symptoms rather than symptom clusters, enabling identification of specific symptom-level associations between PTSS and depressive symptom communities. Third, we applied DSM-5–based PTSS criteria for PTSD assessment and depressive symptom measures relevant to major depressive disorder, including negative alterations in mood that overlap with depressive-like symptoms. Because network analysis is data-driven, the inclusion of DSM-5–aligned symptoms may yield particularly informative insights into symptom structure.

Several limitations of this study should be noted. First, the repeated cross-sectional design, rather than a panel design, limits inference regarding dynamic symptom changes and causality within the estimated networks; future research should apply longitudinal and multilevel modeling approaches to address symptom trajectories and prognosis. Second, the findings may not generalize to clinical populations, such as individuals receiving treatment for PTSD. Third, sleep disturbance was assessed using single-item measures rather than multidimensional, validated instruments, such as the Pittsburgh Sleep Quality Index. Fourth, although the sample was population-based, it was racially homogeneous and limited to adults; future studies should examine comorbid PTSS–depression network structures in more diverse and life-course–representative samples. Fifth, due to sample size and network stability constraints, subgroup analyses by demographic characteristics, such as gender or age, could not be conducted. Future research should evaluate whether network structures differ across demographic subpopulations.

During the subacute and chronic phases of the COVID-19 pandemic in Korea, we found evidence of changes in the network structure of PTSS and depressive symptoms, even as sleep problems consistently functioned as a bridging symptom. These findings suggest that, during future pandemics, prompt screening and early identification of sleep problems may help prevent the development of comorbidity between PTSS and depressive symptoms.

## Figures and Tables

**Figure f5-epih-48-e2026006:**
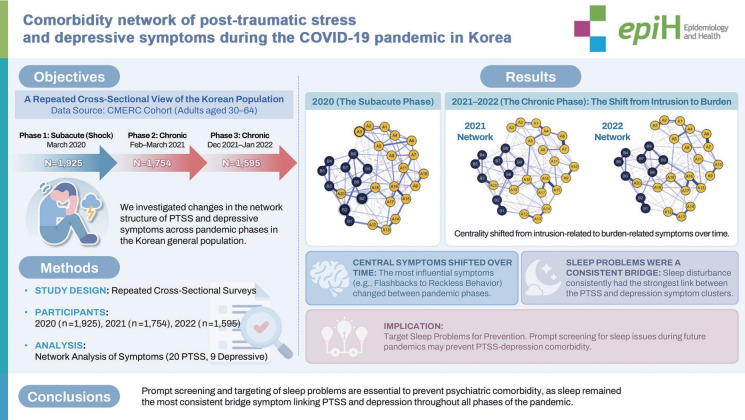


**Table 1.** PHQ-9 and PCL-5 scores throughout the COVID-19 pandemic by gender^1^

Characteristics	Total (n=1,970)	Men (n=693)	Women (n=1,277)	p-value
Age (yr)	55.49±9.24	55.20±9.90	55.65±8.78	<0.01
Smoking				<0.01
Never	1,383 (70.2)	187 (27.0)	1,196 (93.7)	
Past	363 (18.4)	315 (45.5)	48 (3.8)	
Current	224 (11.4)	191 (27.6)	33 (2.6)	
Drinking				<0.01
Never	412 (20.9)	60 (8.7)	352 (27.6)	
Past	91 (4.6)	45 (6.5)	46 (3.6)	
Current	1,467 (74.5)	588 (84.9)	879 (68.8)	
Regular exercise (min/wk)				<0.01
Low (0)	914 (46.4)	276 (39.8)	638 (50.0)	
Middle (<150)	251 (12.7)	88 (12.7)	163 (12.8)	
High (≥150)	805 (40.9)	329 (47.5)	476 (37.3)	
Marital status				<0.01
Never married	117 (5.9)	54 (7.8)	63 (4.9)	
Living together	1,705 (86.6)	627 (90.5)	1,078 (84.4)	
Living alone	18 (0.9)	4 (0.6)	14 (1.1)	
Divorced or widowed	130 (6.6)	8 (1.2)	122 (9.6)	
Length of formal education (yr)				<0.01
≤6	54 (2.7)	9 (1.3)	45 (3.5)	
≤9	121 (6.1)	26 (3.8)	95 (7.4)	
≤12	691 (35.1)	190 (27.4)	501 (39.2)	
>12	1,104 (56.0)	468 (67.5)	636 (49.8)	
Household income				<0.01
Q1	403 (20.5)	105 (15.2)	298 (23.3)	
Q2	643 (32.6)	231 (33.3)	412 (32.3)	
Q3	372 (18.9)	142 (20.5)	230 (18.0)	
Q4	552 (28.0)	215 (31.0)	337 (26.4)	
Disease history				0.41
No	1,116 (56.7)	384 (55.4)	732 (57.3)	
Yes	854 (43.4)	309 (44.6)	545 (42.7)	
Current medication intake				0.05
No	1,288 (65.4)	433 (62.5)	855 (67.0)	
Yes	682 (34.6)	260 (37.5)	422 (33.1)	
PHQ-9 scores				
2020 survey	2.72±3.71	2.83±3.82	2.66±3.70	0.34
2021 survey	4.92±5.02	4.08±4.78	5.35±5.04	<0.01
2022 survey	4.52±4.87	3.49±4.40	5.06±5.02	<0.01
PCL-5 scores				
2020 survey	10.29±10.20	10.25±10.02	10.31±10.37	0.21
2021 survey	9.68±11.72	8.68±11.36	10.20±11.91	0.02
2022 survey	10.42±12.60	9.06±11.90	11.16±12.90	0.01

Values are presented as mean±standard deviation or number (%). PHQ-9, Patient Health Questionnaire-9; PCL-5, Post-traumatic Stress Disorder Checklist for the Diagnostic and Statistical Manual of Mental Disorders, fifth edition; COVID-19, coronavirus disease 2019.

1 Sum of numbers may not reflect the total number in the group due to missing values.

**Table 2.** Changing patterns of symptom ranking (highest metrics) throughout the COVID-19 pandemic

Ranking	Subacute phase		Chronic phase			
	2020 Survey		2021 Survey		2022 Survey	
	Community	Node (index)	Community	Node (index)	Community	Node (index)
Central symptom^1^						
1	PHQ-9	Depressed mood (2.35)	PHQ-9	Depressed mood (2.22)	PHQ-9	Depressed mood (1.98)
2	PCL-5	Flashbacks (2.24)	PCL-5	Reckless behavior (1.88)	PCL-5	Reckless behavior (1.79)
Bridge central symptom^2^						
1	PCL-5	Sleeping disturbance (2.04)	PHQ-9	Sleeping problems (1.96)	PHQ-9	Sleeping problems (2.97)
2	PHQ-9	Sleeping problems (1.97)	PCL-5	Sleeping disturbance (1.87)	PCL-5	Sleeping disturbance (2.32)

COVID-19, coronavirus disease 2019; PHQ-9, Patient Health Questionnaire-9; PCL-5, Post-traumatic Stress Disorder Checklist for the Diagnostic and Statistical Manual of Mental Disorders, fifth edition; EI, expected influence.

1 The central symptom was calculated using the EI.

2 The bridge central symptom was calculated using the bridge EI.

**Table 3.** Summary of A20–B3 edge weights and strongest cross-group associations across 2020–2022 surveys^1^

Year	A20–B3 weight	Rank among all A–B edges	Strongest connections	
			From A20 to B symptoms	From B3 to A symptoms
2020	0.38	1st	B3 (0.38), B9 (0.04), B6 (0.02)	A20 (0.38), A15 (0.04), A3 (0.02)
2021	0.45	1st	B3 (0.45), B9 (0.07), B6 (0.05)	A20 (0.45), A12 (0.03), A19 (0.03)
2022	0.44	1st	B3 (0.44), B1 (0.10), B9 (0.06)	A20 (0.44), A17 (0.09), A19 (0.05)

A20, sleep disturbance; B3, sleeping problems.

1 Higher absolute edge weights indicate stronger conditional dependence between symptoms.

## Supplementary materials

Supplementary Material 1. The trend of daily new cases and cumulative cases of COVID-19 in South Korea and the time points of the CC-MHS.

Supplementary Material 2. Correlation matrix of edge weight in a comorbidity network of the 2020 survey.

Supplementary Material 3. Correlation matrix of edge weight in a comorbidity network of the 2021 survey.

Supplementary Material 4. Correlation matrix of edge weight in a comorbidity network of the 2022 survey.

Supplementary Material 5. Bootstrapped confidence intervals of estimated edge weights for the comorbidity network.

Supplementary Material 6. Stability of both expected influence and bridge expected influence.
