# Supplementary material for: Comorbidity network of post-traumatic stress and depressive symptoms during the COVID-19 pandemic in Korea
Source: Epidemiol Health. 2026 Jan 23;48:e2026006. doi: 10.4178/epih.e2026006 (PMC13034016; doi:10.4178/epih.e2026006)

**Supplementary Material 1. The trend of daily new cases and cumulative cases of COVID-19 in South Korea and the time points of the CC-MHS.**


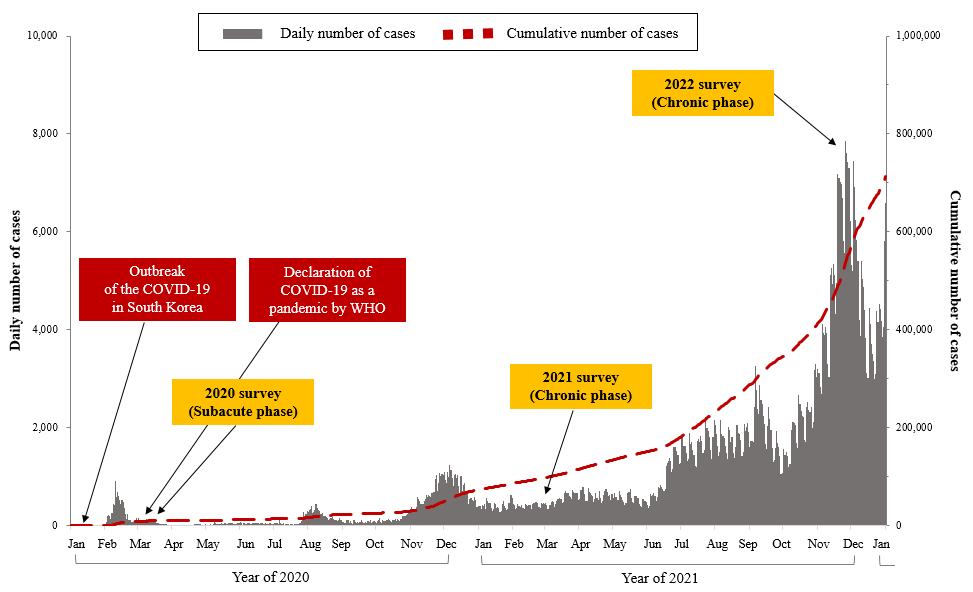

Supplement: Supplementary Material 1. — The trend of daily new cases and cumulative cases of COVID-19 in South Korea and the time points of the CC-MHS. [file epih-48-e2026006-Supplementary-1.docx]
