# Supplementary material for: Comorbidity network of post-traumatic stress and depressive symptoms during the COVID-19 pandemic in Korea
Source: Epidemiol Health. 2026 Jan 23;48:e2026006. doi: 10.4178/epih.e2026006 (PMC13034016; doi:10.4178/epih.e2026006)
Supplement: Supplementary Material 4. — Correlation matrix of edge weight in a comorbidity network of the 2022 survey. [file epih-48-e2026006-Supplementary-4.docx]

Supplementary Material 4. Correlation matrix of edge weight in a comorbidity network of the 2022 survey.

**Notes.** The blue-color box shows a positive correlation between symptoms, while the red-color box shows a negative correlation. Bold means a correlation of the highest several of the edge weights. **A1-A20 are items of PCL-5**: A1:intrusive thoughts; A2: Nightmares; A3: Flashbacks; A4: Emotional cue reactivity; A5: Physiological cue reactivity; A6: Avoidance of thoughts; A7: Avoidance of reminders; A8: Trauma-related amnesia; A9: Negative beliefs; A10: Blame of self or others; A11: Negative trauma-related emotions; A12: Loss of interest; A13: Detachment; A14: Restricted affect; A15: Irritability/anger; A16: Reckless/self-destructive behavior; A17: Hypervigilance; A18: Exaggerated startle response; A19: Difficulty concentrating; A20: Sleep disturbance. **B1-B9 are items of PHQ-9:** B1: Anhedonia; B2: Depressed mood; B3; Sleeping problems; B4: Fatigability; B5: Appetite problems; B6: Negative feeling by myself; B7: Concentration problems; B8: Agitation/retardation; B9: Suicidal ideation.
