# Supplementary material for: Comorbidity network of post-traumatic stress and depressive symptoms during the COVID-19 pandemic in Korea
Source: Epidemiol Health. 2026 Jan 23;48:e2026006. doi: 10.4178/epih.e2026006 (PMC13034016; doi:10.4178/epih.e2026006)
Supplement: Supplementary Material 5. — Bootstrapped confidence intervals of estimated edge weights for the comorbidity network. [file epih-48-e2026006-Supplementary-5.docx]

**Supplementary Material 5. Bootstrapped confidence intervals of estimated edge weights for the comorbidity network.** (a) Accuracy test of the 2020 survey, (b) Accuracy test of the 2021 survey, (c) Accuracy test of the 2022 survey.


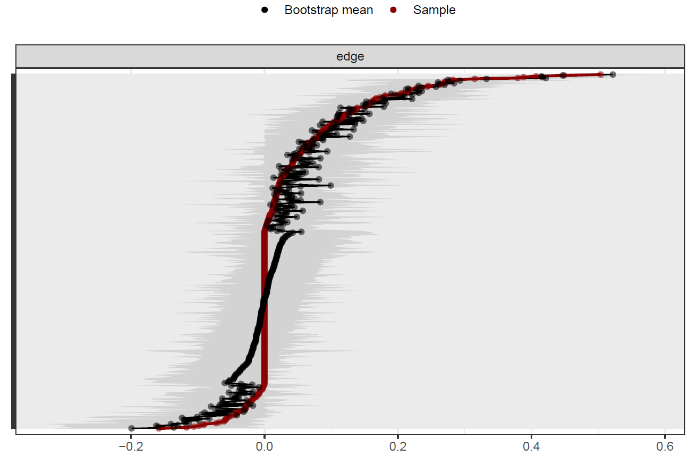


(a)


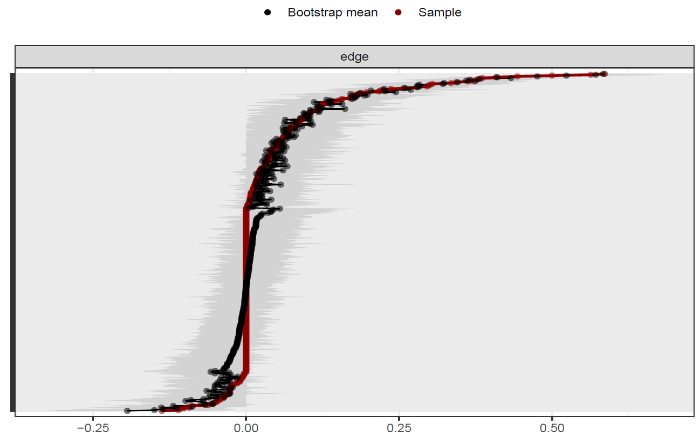

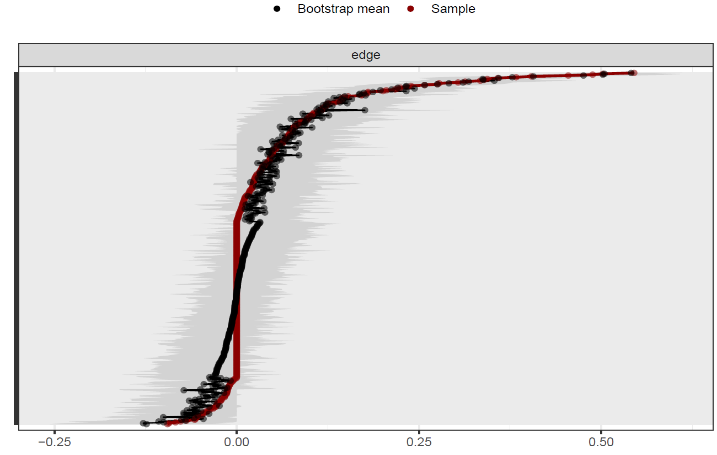


(c)

(b)
